# We were wrong on the benefits of the extra-cardiac Fontan. Should we go back to the lateral tunnel?

**DOI:** 10.1093/icvts/ivad187

**Published:** 2023-11-22

**Authors:** Yves d’Udekem, Eiri Kisamori, Can Yerebakan

**Affiliations:** Division of Cardiovascular Surgery, Children’s National Heart Institute, Children’s National Hospital, Washington, DC, USA; Division of Cardiovascular Surgery, Children’s National Heart Institute, Children’s National Hospital, Washington, DC, USA; Division of Cardiovascular Surgery, Children’s National Heart Institute, Children’s National Hospital, Washington, DC, USA

**Keywords:** Univentricular heart disease, Fontan circulation, Lateral tunnel Fontan, Extracardiac conduit Fontan, Conduit size

The team of Leiden present 1 more work demonstrating the lack of adequacy of small-size extracardiac conduits for the Fontan completion [1]. Through complex computational fluid dynamics studies, correlated by real-life measurement of flows on exercise, they already demonstrated that the ideal size of the conduit should be between 22 and 28 mm in an adult to provide if we want these adults to benefit from the best possible exercise capacity [2]. In the current work, in a virtual intervention study, they analyse the extent of the benefits that patients with 16-mm extra-cardiac conduits could have if they were dilated to 3 different sizes.

Unsurprisingly, they propose that if the patients have larger conduits, they will have improved flows and smaller resistances and we can expect them to have a better exercise capacity.

This team is now pointing to a potentially enormous issue. In the beginning of the century, our community has started to perform less lateral and more extra-cardiac Fontan, so that the latter has become clearly the most frequently performed operation [3]. We are now finding the hard way that the extracardiac may not have fulfilled its promises and may carry additional unexpected complications. The extra-cardiac Fontan gained its current popularity because we all thought that reducing the amount of intracardiac suture lines would protect patients against the occurrence of the frequently observed arrhythmias but, 3 decades later, this hope does not seem to have been fulfilled [4]. While there are some discrepant observations, it seems that there is not much difference between the 2 procedures in terms of arrythmias.

Many teams had advocated for the use of smaller size conduits when operating children under 3 years of age as larger size conduits seemed to bring no benefits and even be inferior to small ones. A study focusing on patients with a mean body surface area of 0.53 m^2^ demonstrated that larger conduits had redundant spaces, suggesting that 16- and 18-mm conduits were the optimal choices [5].

The team from Leiden, as others, demonstrate the lack of suitability of these small conduits for optimal exercise capacity. Another emerging major issue is the observed increased incidence of liver fibrosis and cirrhosis in patients implanted with small conduits. In 2018, in an in-depth cross-sectional study, we demonstrated that patients with extra-cardiac conduits had clearly more liver fibrosis than their lateral tunnel counterpart [6]. Our preliminary review of North American data seems to confirm this fact. Over the last year, we have started a new procedure: the upsizing of small Fontan conduits. Four patients were operated, and the main indication was rapidly progressing liver cirrhosis and gastrointestinal bleeding in 2. Fortunately, these procedures seemed to be well tolerated. In our team in Washington, DC, we have no doubt that all of the patients implanted with 14- and 16-mm conduits will need to undergo a reoperation. Larger size conduits may be offered dilatation and stenting.

But what are we going to do with those with 18- and 20-mm conduits? The extra-cardiac conduit may lead to higher degree of cirrhosis which may impact the longevity of these patients in their final decades of life. Will we wait the 2 decades that will be necessary to prove that fact to intervene?

We believe that it is now time to revive the lateral tunnel procedure, at least in the small patients. It is noteworthy that the lateral tunnel pathway of Fontan is known to exhibit growth over time [7]. There are now new grafts that allow balloon expansion without stenting that are now being investigated and this is an interesting avenue to explore. Those who stick to the extra-cardiac conduit Fontan should understand that this procedure will necessitate other reinterventions before an adult size is reached.
